# Measuring recent in-country TB transmission using a classification model with whole genome sequencing data

**DOI:** 10.5588/ijtldopen.25.0735

**Published:** 2026-05-11

**Authors:** W. Frederiks, R.M. Anthony, G. de Vries

**Affiliations:** 1 Centre for Infectious Diseases, Epidemiology and Surveillance, National Institute for Public Health and the Environment, Bilthoven, the Netherlands;; 2 National Tuberculosis Reference Laboratory, Centre for Infectious Disease Research, Diagnostics and Laboratory Surveillance, National Institute for Public Health and the Environment, Bilthoven, the Netherlands;; 3 National Coordination Centre for Communicable Disease Control, National Institute for Public Health and the Environment, Bilthoven, the Netherlands.

**Keywords:** tuberculosis, WGS, the Netherlands, TB infection, TB pre-elimination

## Abstract

**BACKGROUND:**

Molecular typing of *Mycobacterium tuberculosis* isolates provides insight into TB transmission by identifying clustering isolates. However, clustering alone does not necessarily indicate ongoing in-country transmission, as infections may have occurred abroad or long ago.

**METHODS:**

We developed a classification model categorising clustering TB patients into three groups: i) likely, ii) possibly, and iii) unlikely to have been recently infected in the Netherlands. The model consists of two stages: i) individual labelling, followed by ii) cluster labelling for the remaining cases, and includes epidemiological, whole genome sequencing (WGS) data, and confirmed epidemiological links based on cluster investigation.

**RESULTS:**

We found that 28% of TB patients with WGS results in 2018–2023 had a clustered isolate. However, when we classified cases by individual labelling (48% of cases) and cluster labelling (52% of cases), only 11% to a maximum of 15% were likely to have been infected within the last 2 years in the Netherlands.

**CONCLUSION:**

Our WGS-based classification model provides a valuable tool for monitoring progress towards TB pre-elimination by enabling estimation of recent in-country transmission. Importantly, our findings indicate that a substantial proportion of clustered cases were likely the result of infections acquired abroad or from non-recent transmission events.

The WHO has set ambitious targets to end TB, aiming to reduce global TB incidence by 90% compared to 2015-levels and to achieve fewer than 10 cases per 100,000 population by 2035.^1^ Countries that have already reached this low-incidence threshold are now focusing on pre-elimination (fewer than 1 case per 100,000 population).^2^ In 2014, the WHO and the European Respiratory Society developed an action framework for TB elimination in low-incidence countries, outlining eight core areas to support progress towards pre-elimination.^2,3^ More recently, Migliori et al.^4^ proposed a five-stage framework to guide TB elimination efforts, including disease reduction targets every 5 years, alongside priority actions and key interventions. The Netherlands is a low TB-incidence country, reporting a notification rate of 4.0 per 100,000 population in 2023, down from 5.1 per 100,000 in 2015.^5^ In 2023, 82% of TB cases were in people born outside the Netherlands. The notification rate among native-born individuals was 0.9 per 100,000, already below the pre-elimination threshold, while the rate among foreign-born individuals was 20.3 per 100,000.

Since 2011, the Netherlands has implemented 5-year national strategic plans for TB control (TB-NSPs).^6^ The TB-NSPs for 2016–2020 and 2021–2025 aim to reduce the TB burden by 25% every 5 years, in alignment with WHO targets. Recognising that external factors such as migration influence overall incidence, a second more relevant key indicator was introduced: reducing transmission within the Netherlands by 25% every 5 years. To monitor transmission trends within the Netherlands, two methods have been proposed: i) assessing the number of TB cases attributed to recent in-country transmission using molecular typing and ii) evaluating trends in individuals with TB and TB infection identified through contact investigations of infectious pulmonary TB patients.^7^

Universal molecular typing of all *Mycobacterium tuberculosis* strains has been conducted in the Netherlands since 1993, initially with restriction fragment length polymorphism (RFLP) followed by variable number of tandem repeats (VNTR). In 2018, whole genome sequencing (WGS), which has higher typing resolution, replaced VNTR.^8,9^ Molecular clustering alone however does not necessarily indicate ongoing in-country transmission, as infections may have occurred abroad or long ago.^10^ Therefore, methods have been applied to distinguish recent transmission from genotypic clustering, incorporating factors such as the geographic proximity between linked cases.^11–14^ In the Netherlands, public health TB nurses systematically investigate epidemiological links between recently clustered cases (cluster investigation).^15^ Previously, a highly detailed transmission classification model was developed for one area in the Netherlands to categorise molecularly clustered patients as i) recently (<2 years) or ii) remotely (≥2 years) infected within the Netherlands, or iii) infected in a foreign country, based on molecular typing and epidemiological data.^16^ In our study, we aimed to develop a more generalisable classification model applicable at the national level, fulfilling the requirements needed to monitor the second key indicator of the Dutch TB-NSP (reducing transmission *within* the Netherlands by 25% every 5 years).

## METHODS

TB is a notifiable disease in the Netherlands, and all diagnosed cases must be reported to the Netherlands Tuberculosis Register (NTR). For this study, we included patients notified to the NTR between 2018 and 2023. We retrieved NTR data on patient sex, age, country of birth (CoB), duration of residence in the Netherlands at diagnosis, and WGS results, including confirmation of epidemiological links identified through cluster investigation. Additional WGS data from patients diagnosed and notified during 2016–2017 were also obtained to classify clustering and determine cluster characteristics.

### DNA fingerprinting and definitions

Patients with isolates differing by ≤12 single nucleotide polymorphisms (SNPs) were considered clustered,^17^ except for the first case in a cluster, which was classified as unique according to the ‘n − 1 method’.^18^

### Current transmission model

For each clustered patient, the time interval since the previous case was assessed: if the preceding case occurred <2 years ago, it was defined as recent clustering; if ≥2 years ago, it was defined as non-recent clustering.^16^ Patients whose isolates did not cluster with any other isolate in the entire 2016–2023 dataset were classified as unique. In the Netherlands, an epidemiological link is ‘confirmed’ when cluster investigation determines that two individuals within the same cluster were simultaneously present at a shared location, often indicating they knew each other.

### Transmission classification model

To measure recent transmission in the Netherlands, we developed a classification model categorising clustered TB patients into three groups: i) likely, ii) possibly, and iii) unlikely to have been recently infected in the Netherlands. The model consists of two stages: i) individual labelling, followed by ii) cluster labelling. An overview is provided in Table 1.

**Table 1.** TB transmission classification model.

Individual labelling		
Arguments	Label
1. Epidemiological link	Likely infected in the Netherlands
2. <3 months in the Netherlands at diagnosis	Likely infected in the Netherlands
3. Born in the Netherlands	Unlikely infected in the Netherlands

Cluster labelling – Scoring system		
Arguments	Points	Weights
Added points for arguments for transmission in the Netherlands		
- Epidemiological link	number/n – index	5
- Born in the Netherlands	number/n	1
- Multiple countries of birth in cluster	number/n	1
Subtracted points for arguments against transmission in the Netherlands		
- <3 months in the Netherlands at diagnosis	number/n – index	1
- One country of birth in cluster other than the Netherlands	1	1
$Percentage\;cluster\;score=\left(\;\frac{Cluster\;score+2}{\;Maximum\;achievable\;cluster\;score+2}\;\right)\times 100$		

Cluster labelling – Labelling	
Percentage cluster score	Label
<26%	Unlikely infected in the Netherlands
26%–46%	Possibly infected in the Netherlands
>46%	Likely infected in the Netherlands

‘number’ refers to the number of patients for which an argument is fulfilled. n = total number of patients within a cluster.

### Individual labelling

Three hierarchical criteria were applied to classify clustering patients at the individual level:

1. Patients with a confirmed epidemiological link were classified as likely infected in the Netherlands.
2. Patients who had been in the Netherlands for less than 3 months at diagnosis were classified as unlikely infected in the Netherlands, based on the minimal incubation period of TB.^11^
3. Native-born patients were classified as likely infected in the Netherlands, assuming a high probability that infection occurred from a circulating strain within the country.

### Cluster labelling

For clustering patients who could not be individually labelled, we developed a scoring system consisting of non-hierarchical criteria estimating the likelihood that their infection resulted from transmission in the Netherlands. Criteria supporting in-country transmission included:

- Presence of a confirmed epidemiological link in the Netherlands for at least one pair of isolates within the cluster;
- Patients born in the Netherlands within the cluster;
- Presence of multiple CoBs within the cluster. Multiple CoBs suggest in-country transmission, as it is unlikely that these patients from diverse origins were infected abroad by the same strain.

Criteria against transmission in the Netherlands included:

- Having been in the Netherlands for less than 3 months at diagnosis.
- Presence of a single CoB different from the Netherlands in the entire cluster. A single foreign CoB suggests infection in the patients’ country of birth or during shared travel to the Netherlands, as was described in an outbreak involving patients from the Horn of Africa.^19^ This is particularly relevant in the Netherlands, since a large proportion of foreign-born TB patients originate from the Horn of Africa.^5^ Eritrea and Ethiopia were considered the same CoB in this analysis due to their shared history until 1993.

### Scoring system

For each criterion, points were assigned and weighted relative to the predictive value of other criteria. Weights were determined through a modified Delphi method with TB experts who independently completed a questionnaire over two rounds. A percentage cluster score ranging from 0% to 100% was then calculated for patients not individually labelled, with higher scores indicating greater probability of transmission within the Netherlands. The detailed calculation is described in the Supplementary Data. Cluster score thresholds were then determined to classify the not-individually labelled cases as i) likely, ii) possibly, or iii) unlikely infected in the Netherlands.

### Recent transmission in the Netherlands

Recently clustered patients classified as likely being infected in the Netherlands were considered recent in-country transmission cases. Those recently clustered and classified as possibly infected in the Netherlands were included to estimate the maximum percentage of recent in-country transmission.

### Ethical statement

The Registration Committee of the NTR approved the study proposal and provided anonymised data.

## RESULTS

**Figure 1.**
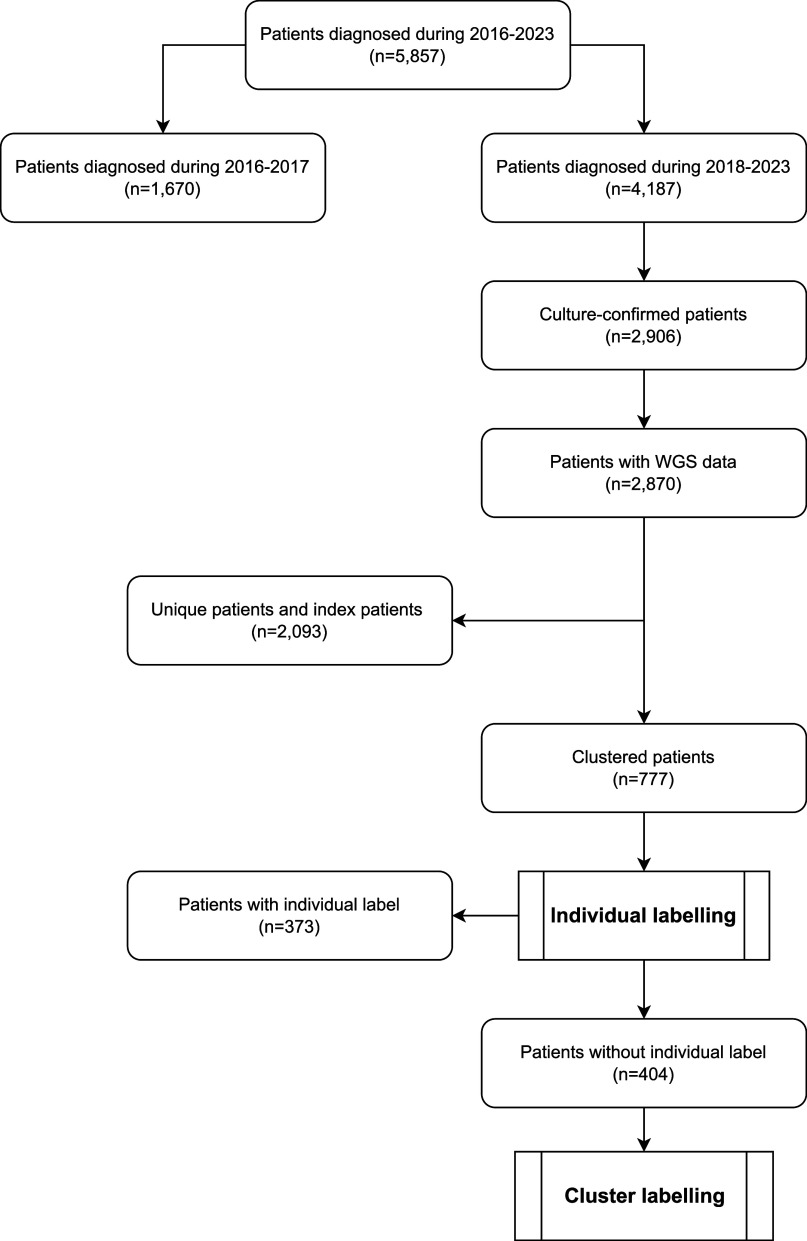
Flow chart of TB patients notified in the Netherlands, 2016–2023. WGS = whole genome sequencing.

Between 2018 and 2023, a total of 4,187 TB cases were registered in the Netherlands, of which 2,906 (69%) were culture-confirmed (Figure 1). WGS was performed for 2,870 cases, representing 99% of the culture-confirmed cases. Of these, 2,093 (72.9%) had a unique WGS profile or were the first case in a cluster, while 777 (27.1%) clustered with at least one other (previous) isolate. Among the 777 clustered cases, 623 (80.2%) were classified as recently clustered, and 154 (19.8%) as non-recently clustered.

### Classification by individual labelling

Among the 777 clustered patients, 192 (24.7%) had a confirmed epidemiological link, 49 (6.3%) had been in the Netherlands <3 months at the time of diagnosis (48.0% in total), and 132 (17.0%) were born in the Netherlands. Based on these criteria, 324 patients were classified as likely infected in the Netherlands and 49 as unlikely infected in the Netherlands. The remaining 404 patients (52.0%) could not be individually classified and were therefore classified using cluster labelling.

### Classification by cluster labelling

**Figure 2.**
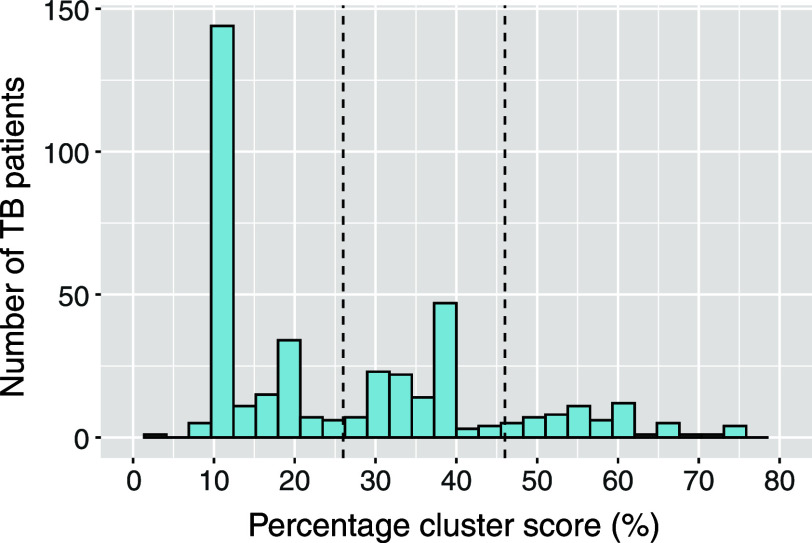
Distribution of percentage cluster scores of not-individually labelled clustered TB patients in the Netherlands, 2018–2023. The distribution of percentage cluster scores of all 404 not-individually labelled clustered patients. The two vertical dashed lines indicate the thresholds of the percentage cluster scores at 26% and 46%. The distributions of percentage cluster scores of the not-individually labelled clustered patients in small (n ≤ 3) and large (n > 3) clusters are shown in Supplementary Data Figure S1.

Using the modified Delphi approach, the presence of an epidemiological link was assigned a weight of 5, while all other criteria were assigned a weight of 1 (Table 1). The distribution of percentage cluster scores is shown in Figure 2, with small (n ≤ 3) and large (n > 3) clusters displayed separately in Supplementary Data Figure S1. For small clusters, groups of patients with similar percentage cluster scores are clearly distinguishable; this distinction is more challenging in large clusters. Based on the distribution of percentage cluster scores and expert panel classification, thresholds were set at a percentage cluster score of 26% and 46% to distinguish between patients unlikely (<26%), possibly (26%–46%), or likely (>46%) infected in the Netherlands. Among the 404 patients assessed through cluster labelling, 223 were classified as unlikely, 120 as possibly, and 61 as likely infected in the Netherlands. Among those classified as unlikely infected in the Netherlands, 136 (61.0%) were born in Eritrea or Ethiopia.

### Overall classification and trends

**Figure 3.**
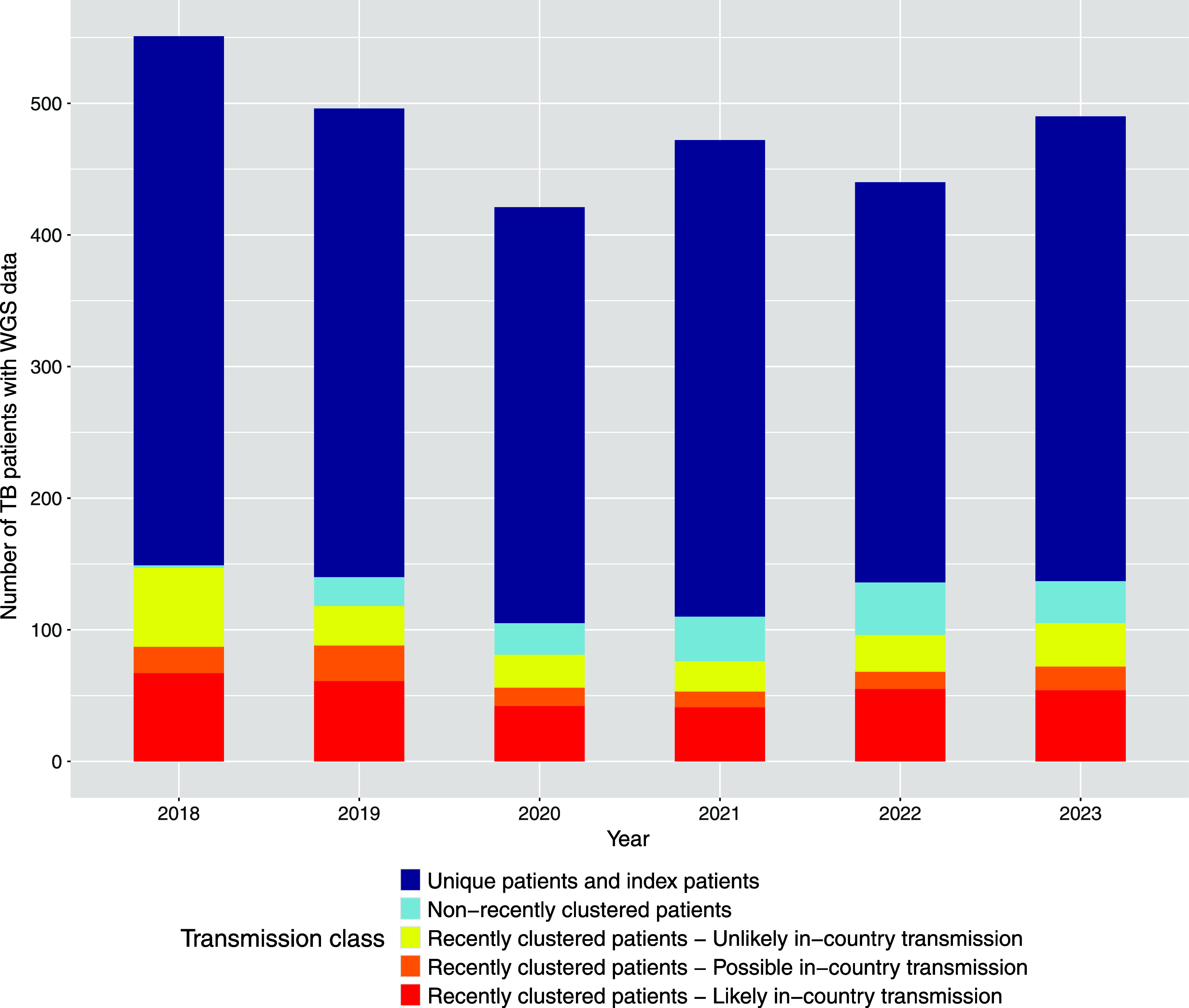
Distribution of TB patients notified in the Netherlands with whole genome sequencing data, 2018–2023, per year and transmission class of the classification model. The proportional distribution of TB patients across the transmission classes of the classification model is provided in Supplementary Data Figure S2.

During 2018–2023, 11% of WGS-genotyped patients were due to recent transmission within the Netherlands, increasing to a maximum of 15% when patients possibly infected in the Netherlands were included (Table 2). We observed a decline in the percentage of recent in-country transmission from 12% in 2019 to 9% in 2021, coinciding with the COVID-19 pandemic. This was followed by a rebound to 13% in 2022 and 11% in 2023. Bar plots showing the absolute and proportional distribution of WGS-fingerprinted patients across classification categories are presented in Figure 3 and Supplementary Data Figure S2. Culture-negative patients, comprising 31% of notified TB cases in the Netherlands (Figure 2), were excluded from the analysis due to the absence of WGS data. Among these patients, 9.9% had been in the Netherlands <3 months at the time of diagnosis and 12.0% were identified through contact investigation (Supplementary Data Table S1).

**Table 2.** Number of TB patients notified in the Netherlands with whole genome sequencing (WGS) data, 2018–2023, per year and transmission class of the classification model.

Transmission class	2018	2019	2020	2021	2022	2023	Total
TB patients with WGS data (%)	551 (100)	496 (100)	421 (100)	472 (100)	440 (100)	490 (100)	2,870 (100)
Unique patients and index patients (%)	402 (73)	356 (72)	316 (75)	362 (77)	304 (69)	353 (72)	2,093 (73)
Clustered patients (%)	149 (27)	140 (28)	105 (25)	110 (23)	136 (31)	137 (28)	777 (27)
Non-recently clustered patients (%)	2 (0)	22 (4)	24 (6)	34 (7)	40 (9)	32 (7)	154 (5)
Recently clustered patients – unlikely in-country transmission (%)	60 (11)	30 (6)	25 (6)	23 (5)	28 (6)	33 (7)	199 (7)
Recently clustered patients – possible in-country transmission (%)	20 (4)	27 (5)	14 (3)	12 (3)	13 (3)	18 (4)	104 (4)
Recently clustered patients – likely in-country transmission (%)	67 (12)	61 (12)	42 (10)	41 (9)	55 (13)	54 (11)	320 (11)

## DISCUSSION

We developed and applied a classification model that integrates WGS and epidemiological data to estimate recent in-country TB transmission. Using national surveillance data, we found that 28% of TB patients with WGS results in 2018–2023 had a clustered isolate. However, when we classified cases by individual labelling (48% of cases) and cluster labelling (the remaining 52% of cases), only 11% to maximum of 15% were likely to have been recently infected within the Netherlands. Understanding the proportion of cases resulting from recent in-country transmission is important for monitoring the effect of and guiding TB control efforts. When transmission mainly occurs outside the country, cross-border TB control activities should be strengthened,^20,21^ whereas a high proportion of in-country transmission may warrant intensified contact investigation efforts. In the Netherlands, the majority of TB cases are in foreign-born persons who acquired the infection prior to arrival.^5,22^ Consequently, prevention efforts including screening for TB disease on arrival, and more recently also screening for TB infection, are offered in the Netherlands to migrants and asylum seekers from priority countries.^23–27^

Previous studies estimating recent TB transmission mainly differed in the genotyping method applied (RFLP or VNTR rather than WGS), as well as the methods for distinguishing in-country transmission from transmission outside the country, often utilising spatial data. Moonan et al.^28^ analysed VNTR-typed TB cases in the USA and found that the proportion of clustering varied by geographical scale: 77.3% at the national level, 57.1% at the state level, and 38.7% at the county level. They defined recent transmission as cases with matching genotypes occurring within a 50 km radius and a 3-year time window, and estimated that 23.1% of clustered cases were due to recent transmission. France et al.^11^ proposed identifying recent transmission as cases diagnosed within 2 years of a respiratory TB case (or 3 months prior to the putative source case), sharing a similar RFLP-based genotype, and residing within 10 miles (16 km). As in our study, clustered cases diagnosed shortly after arrival were not considered to result from in-country transmission. Using this method, they estimated that 11.4% of genotyped cases, in the four US states studied, were due to recent transmission. Yuen et al.^29^ applied the so-called plausible source-case method from France et al.^11^ to US national data and found that 14% of the VNTR-genotyped cases were attributable to recent transmission. Similarly, a study conducted in the North of England found that 22% of VNTR-genotyped cases were clustered, but using a 3-year temporal window and a spatial window of 50 km only 10% were attributable to localised transmission.^12^

Our study is the first to estimate recent TB transmission using the more precise WGS method. A previous population-based study in the Netherlands demonstrated that the clustering percentages were substantially lower with WGS (14%) than VNTR (25%), while maintaining the same number of identified epidemiological links.^9^ Given the small geographic size of the Netherlands and high population mobility, particularly among people seeking asylum, spatial data were not included in the analysis. Our study has several methodological strengths. It covers a 6-year period and includes nearly all (99%) culture-confirmed TB cases in the Netherlands with available WGS data. Public health teams consistently conducted thorough cluster investigations for clustered cases, and these data were systematically combined and recorded in the National TB Register.

However our study also has limitations. First, culture-negative patients, representing 31% of all notified TB cases in the Netherlands, were excluded from the analysis due to the absence of WGS data. These patients differed from culture-confirmed cases in several important characteristics, including age, disease site (60.5% of culture-negative cases had ETB compared to 31.7% among those with a WGS; Supplementary Table S1), and mode of detection. As such, the transmission estimates presented here are not representative of the entire TB patient population. Second, although confirmed epidemiological links strengthen the evidence for recent in-country transmission, incomplete investigations or undocumented contacts may have led to missed transmission events. Third, some WGS-clustered cases among patients originating from the same country (other than the Netherlands) but without identified epidemiological links may have been misclassified by assuming infection was acquired abroad.

A promising direction for future research is the analysis of newly emerging SNPs within large clusters, which allows further subdivision into sub-clusters and facilitates identification of transmission chains, as recently described.^30^ This approach could enhance the resolution and accuracy of molecular clustering methods.

## CONCLUSION

Our WGS-based classification model provides a valuable tool for monitoring progress towards TB pre-elimination by enabling estimation of recent in-country TB transmission. The model estimates that 11%–15% of molecular-typed cases were attributable to recent transmission within the Netherlands. Importantly, our findings indicate that a substantial proportion of clustered cases were likely the result of infections acquired abroad or from non-recent transmission events. To our knowledge, this is the first study to use epidemiological cluster characteristics to determine whether individual clusters can be explained by in-country transmission. Continued refinement and implementation of such models will be essential to inform public health strategies and accelerate TB elimination efforts.

## Supplementary Material
