# Supplementary material for: Measuring recent in-country TB transmission using a classification model with whole genome sequencing data
Source: IJTLD Open. 2026 May 11;3(5):286–92. doi: 10.5588/ijtldopen.25.0735 (PMC13160269; doi:10.5588/ijtldopen.25.0735)
Supplement: Supplementary file 1 [file ijtldopen25-0735_supplementarydata1.pdf]

Supplement

Scoring system of cluster labelling

Points per argument are calculated by dividing the number of patients in a cluster for which an argument is fulfilled by the total number of patients in that cluster (n). Therefore, clusters that are predominantly explained by transmission in the Netherlands receive more points, whereas clusters in which only a small part or no cases can be explained by transmission in the Netherlands receive less points. Exceptions to this calculation of the points are the arguments about (i) the presence of an epidemiological link, (ii) being in the Netherlands for less than three months at the moment of diagnosis, and (iii) one country of birth in cluster other than the Netherlands.

First, the points scored due to the presence of epidemiological links were calculated as the number of epidemiological links in a cluster divided by the total number of patients in a cluster minus the index case ('number / n – index', Table 1). The index case was removed because one epidemiological link is between two patients. For example, in a cluster of three patients, there can only be a maximum of two epidemiological links. Second, if the index case of a cluster was less than three months in the Netherlands at diagnosis, and therefore likely infected abroad, this person could still have infected all remaining patients within the cluster in the Netherlands. We therefore also removed the index case being less than three months in the Netherlands at diagnosis as an indication for transmission abroad (i.e., number / n – index). Third, the cluster labelling specifies that when a cluster consists of patients who all have the same country of birth (other than the Netherlands), this indicates transmission abroad. Hence, a single point is assigned to the cluster when this argument is fulfilled.

The points scored on each argument were then multiplied by the relative weights determined in the modified-Delphi. Afterwards, a ‘cluster score’ was calculated by adding the points of the arguments for transmission in the Netherlands and subtracting the points of the arguments against transmission in the Netherlands. Subsequently, this cluster score was divided by the ‘maximum achievable cluster score’ and multiplied by 100 to yield a ‘percentage cluster score’. The baseline level of the cluster score was set at the total number of subtraction points (2 points) to produce a more interpretable percentage score between 0 and 100%. A higher percentage cluster score indicates a higher probability that a cluster is mainly explained by in-country transmission. These percentage cluster scores were then assigned to those patients that were not yet labelled during the individual labelling.

Supplementary Figure S1. Distribution of percentage cluster scores of not-individually labelled clustered TB patients in the Netherlands, 2018-2023.

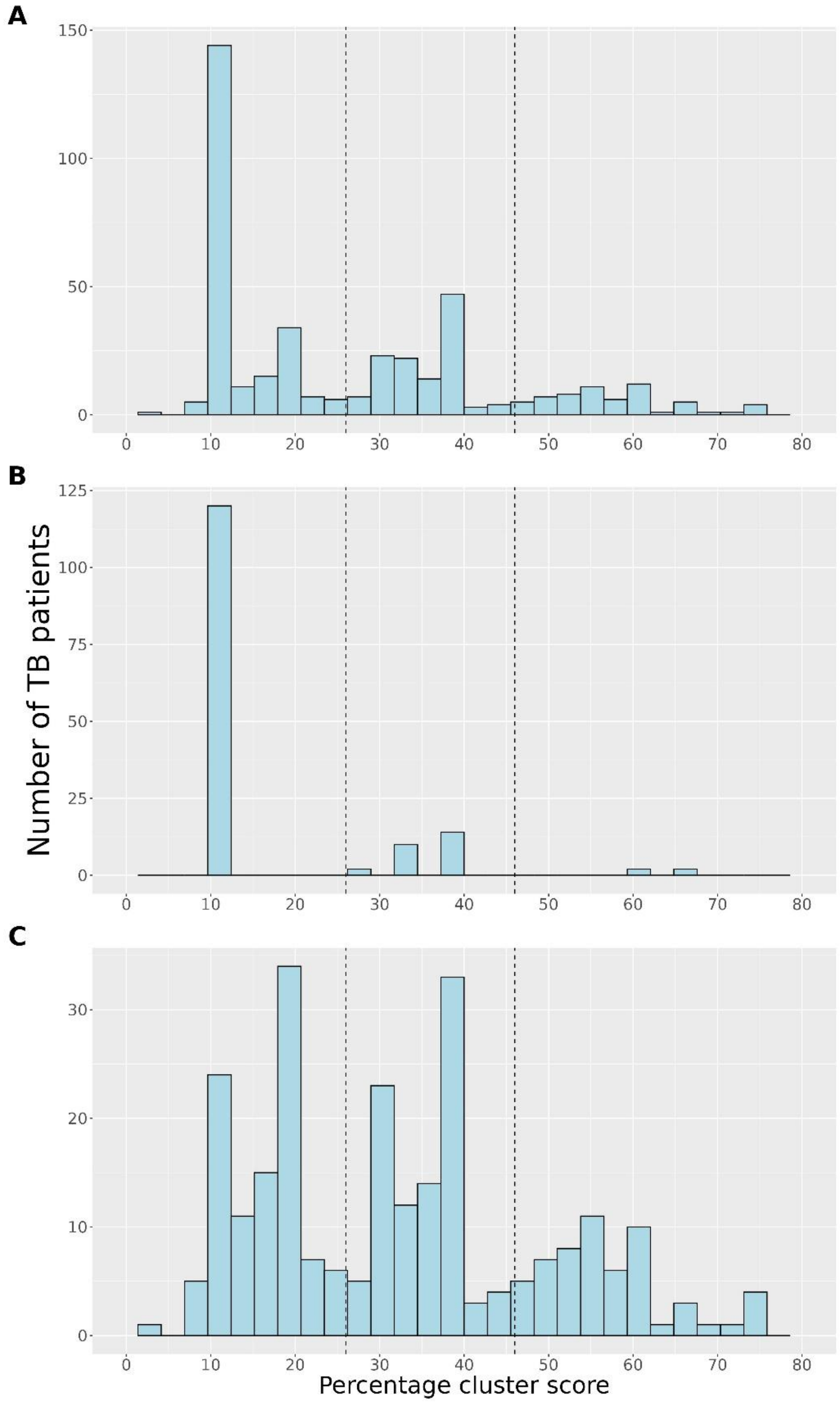

The distributions of percentage cluster scores of all 404 not-individually labelled clustered patients, the 150 patients in small clusters (n ≤ 3) and 254 patients in large clusters (n > 3) are shown in panel A, B and C, respectively. The two vertical dashed lines indicate the thresholds of the percentage cluster scores at 26% and 46%.

Supplementary Figure S2. Absolute (A) and proportional (B) distribution of TB patients notified in the Netherlands with whole genome sequencing data, 2018-2023, per year and transmission class of the classification model.

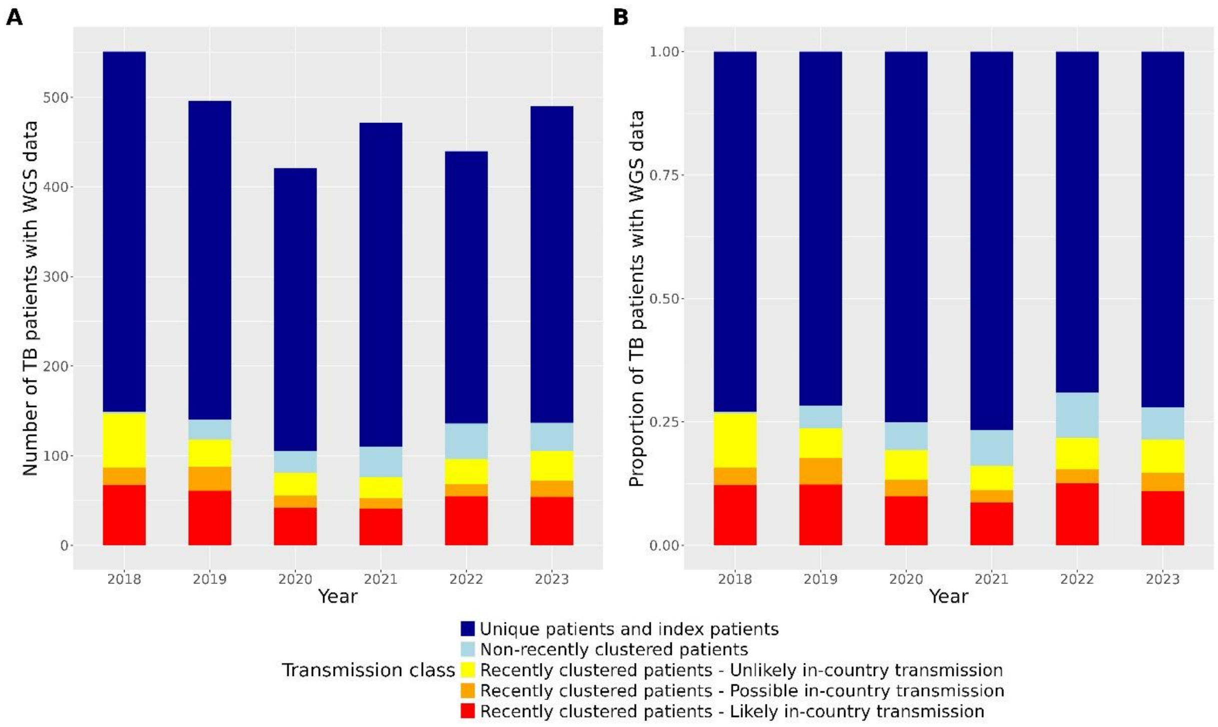

WGS = whole genome sequencing

Supplementary Table S1. Characteristics of TB patients notified in the Netherlands without whole genome sequencing data, 2018-2023.

	Patients without WGS		Patients with WGS		Total			Unique WGS		Clustering WGS	
	n	%	n	%	n	%		n	%	n	%
All cases 2018-2023	1,317	31.5%	2,870	68.5%	4,187	100.0%		2,093	50.0%	777	18.6%
Culture positive	36	2.7%	2,870	100.0%	2,906	69.4%					
Sex											
Male	745	56.6%	1,794	62.5%	2,539	60.6%		1,234	59.0%	560	72.1%
Female	572	43.4%	1,076	37.5%	1,648	39.4%		859	41.0%	217	27.9%
Age group											
0-14 years	110	8.4%	51	1.8%	161	3.8%		23	1.1%	28	3.6%
15-34 years	512	38.9%	385	13.4%	897	21.4%		826	0.0%	385	49.5%
35-64 years	501	38.0%	1,191	41.5%	1,692	40.4%		879	42.0%	312	40.2%
65+ years	194	14.7%	417	14.5%	611	14.6%		365	17.4%	52	6.7%
Country of birth											
Netherlands	352	26.7%	568	19.8%	920	22.0%		338	16.1%	230	29.6%
Foreign country, <90 days in NL	131	9.9%	224	7.8%	355	8.5%		172	8.2%	52	6.7%
Foreign country, ≥90 days in NL or unknown duration of residence	829	62.9%	2,074	72.3%	2,903	69.3%		1,580	75.5%	594	76.4%
Unknown country of birth	5	0.4%	4	0.1%	9	0.2%		3	0.1%	1	0.1%
Type of disease											
Pulmonary TB	520	39.5%	1,960	68.3%	2,480	59.2%		1,355	64.7%	605	77.9%
Extrapulmonary TB	797	60.5%	909	31.7%	1,706	40.7%		738	35.3%	171	22.0%
Case finding method											
Contact investigation	158	12.0%	60	2.1%	218	5.2%		7	0.3%	53	6.8%
Entry screening migrants	145	11.0%	170	5.9%	315	7.5%		129	6.2%	41	5.3%

NL = Netherlands; WGS = Whole genome sequencing
